# *Rabdosia japonica* var. *glaucocalyx* Flavonoids Fraction Attenuates Lipopolysaccharide-Induced Acute Lung Injury in Mice

**DOI:** 10.1155/2014/894515

**Published:** 2014-06-11

**Authors:** Chun-jun Chu, Nai-yu Xu, Xian-lun Li, Long Xia, Jian Zhang, Zhi-tao Liang, Zhong-zhen Zhao, Dao-feng Chen

**Affiliations:** ^1^College of Pharmaceutical Science, Soochow University, Suzhou 215123, China; ^2^School of Chinese Medicine, Hong Kong Baptist University, Kowloon, Hong Kong; ^3^Department of Pharmacognosy, School of Pharmacy, Fudan University, Shanghai 201203, China

## Abstract

*Rabdosia japonica* var. *glaucocalyx* (Maxim.) Hara, belonging to the *Labiatae* family, is widely used as an anti-inflammatory and antitumor drug for the treatment of different inflammations and cancers. *Aim of the Study*. To investigate therapeutic effects and possible mechanism of the flavonoids fraction of *Rabdosia japonica* var. *glaucocalyx* (Maxim.) Hara (RJFs) in acute lung injury (ALI) mice induced by lipopolysaccharide (LPS). *Materials and Methods*. Mice were orally administrated with RJFs (6.4, 12.8, and 25.6 mg/kg) per day for 7 days, consecutively, before LPS challenge. Lung specimens and the bronchoalveolar lavage fluid (BALF) were isolated for histopathological examinations and biochemical analysis. The level of complement 3 (C3) in serum was quantified by a sandwich ELISA kit. *Results*. RJFs significantly attenuated LPS-induced ALI via reducing productions of the level of inflammatory mediators (TNF-**α**, IL-6, and IL-1**β**), and significantly reduced complement deposition with decreasing the level of C3 in serum, which was exhibited together with the lowered myeloperoxidase (MPO) activity and nitric oxide (NO) and protein concentration in BALF. *Conclusions*. RJFs significantly attenuate LPS-induced ALI *via* reducing productions of proinflammatory mediators, decreasing the level of complement, and reducing radicals.

## 1. Introduction

Acute lung injury (ALI) and its more severe form, acute respiratory distress syndrome (ARDS), are the critical pathological condition especially in some severe infectious respiratory diseases [1]. Both of them are characterized by alveolar-capillary membrane disruption, extensive leukocyte infiltration, and releasing of proinflammatory mediators, pulmonary edema associated with proteinaceous alveolar exudates and deterioration of gas exchange, and finally respiratory failure [2]. In addition, the pathogenesis of ALI/ARDS involves the immunity damage and the overactivation of complement system [3]. It reported that complement 3 (C3) is involved in lung injury, and inhibition of complement activation might be a potential therapeutic strategy [4, 5]. Despite recent advances in many new strategies for treatment, the mortality of ALI still remains more than 40% [6]. The development of efficient agents is still urgently needed.

*Rabdosia japonica *var.* glaucocalyx* (Maxim.) Hara, a plant belonging to* genus Rabdosia* in family Labiatae, is used as traditional medicinal herb for centuries in China with low toxicity [7]. In the folk medicine, it is found to be effective in colds, fever, hepatitis, gastritis, mastitis, tonsillitis, liver cancer, and breast cancer [8]. Recently, its modern pharmacological properties were also reported, such as antibacterial and anti-ischemic properties [9, 10]. The main compounds, such as luteolin, quercetin-3-methylether, apigenin, quercetin, luteolin-7-methylether, rutin, isoquercitrin, glaucocalyxin A, glaucocalyxin B, glaucocalyxin C, oleanic acid, and ursolic acid, had been isolated and identified from the herb [11–13].

In our previous study, the ethanol extract of* Rabdosia japonica *var.* glaucocalyx* (Maxim.) Hara was found to contain a lot of flavonoids compounds; furthermore, ten flavonoids were characterized as anticomplementary agents* in vitro* [11–13]. However, whether the flavonoidsfractionof* Rabdosia japonica* var. glaucocalyx (Maxim.) Hara (RJFs) attenuates LPS-induced ALI and the possible mechanisms are still unknown. In the present study, we investigate therapeutic effects and possible mechanism of RJFs on ALI induced by LPS in mice.

## 2. Materials and Methods

### 2.1. Reagents

LPS (*Escherichia coli* 055:B5) was purchased from Sigma-Aldrich Co., Ltd (St. Louis, MO, USA). Dexamethasone (DXM) acetate tablets (number. H33020822) were purchased from Zhejiang Xianju Pharmaceutical Co., Ltd. (Hangzhou, Zhejiang, China). Mouse TNF-*α*, IL-6, and IL-1*β* ELISA kits (number B1007233) were purchased from Shanghai Chuanfu Biotechnology Co., Ltd. (Shanghai, China). Nitric oxide (NO) and bicinchoninic acid (BCA) protein assay kit, myeloperoxidase (MPO), and superoxide dismutase (SOD) determination kits were purchased from Nanjing Jiancheng Bioengineering Institute (Nanjing, Jiangsu, China). The trifluoroacetic acid was purchased from Fluka. *β*-galactosidase and *β*-glucosidase were purchased from Sigma-Aldrich Co., Ltd. Polymyxin B sulfate was purchased from Amresco. Polyclonal rabbit anti-human C3c complement (RS-0367R) was purchased from Shanghai Ruiqi Biological Technology Co., Ltd. (Shanghai, China). The solvents, acetonitrile, and methanol were of HPLC grade from E. Merck (Darmstadt, Germany) and formic acid with a purity of 96% was also in HPLC grade (Tedia, U.S.A.). Water was obtained from a Milli-Q water purification system (Millipore, Bedford, MA, USA). AB-8 macroporous adsorption resins were purchased from Baoen Adsorption-material Technology Co., Ltd (Cangzhou, Hebei, China). All other reagents were of the highest quality available.

### 2.2. Plant Materials and Preparation of RJFs

*Rabdosia japonica* var.* glaucocalyx* (Maxim.) Hara was purchased from Baihe Forest, Taoshan Forestry Administration, Tieli, Heilongjiang Province of China, in October of 2008. The plant material was authenticated by Prof. Zhenyue Wang, Heilongjiang University of Chinese Medicine, and the voucher specimen (number 081008) was deposited at the Herbarium of Materia Medica, Heilongjiang University of Chinese Medicine, Heilongjiang, P. R. China. The extraction and purification of RJFs were carried out according to our previous studies. The dried leaves and stems of* Rabdosia japonica* var.* glaucocalyx* (Maxim.) Hara (100 g) were extracted under reflux with 80% EtOH (1000 mL) for 2 h, repeated twice. After filtration, the combined 80% EtOH extracts were evaporated to dryness under vacuum at 60°C. The solution of extract (0.32 g/mL) was added into column loading-treated AB-8 macroreticular resin for adsorption at 36 mL/min, and then washed with water to get rid of polar impurities. RJFs in the column were eluted with 60% EtOH, and the eluting solution was evaporated to dryness at 60°C until yellow powders were achieved.

### 2.3. Complementary Activity through the Classical Pathway

Based on Mayer's modified method [14], sensitized erythrocytes (EAs) were prepared by incubation of 2% sheep erythrocytes (4.0 × 10^8^ cells/mL) with rabbit anti-sheep erythrocyte antibody (1 : 1000) in VBS^2+^ (containing 0.5 mM Mg^2+^ and 0.15 mM Ca^2+^). Samples were dissolved in VBS^2+^. Guinea pig serum was used as the complement source. The 1 : 60 diluted Guinea pig serum was chosen to give submaximal lysis in the absence of complement inhibitors. In brief, various dilutions of tested samples (200 *μ*L) were mixed with 200 *μ*L of Guinea pig serum, and 200 *μ*L of EAs was added, and then the mixture was incubated at 37°C for 30 min. The different assay controls were incubated in the same conditions: (1) vehicle control: 200 *μ*L EAs in 400 *μ*L VBS^2+^; (2) 100% lysis: 200 *μ*L EAs in 400 *μ*L ultrapure water; (3) samples background: 200 *μ*L dilution of each sample in 400 *μ*L VBS^2+^. The reacted mixture was centrifuged immediately at 4°C after incubation. Optical density of the supernatant was measured at 405 nm on well scan (Labsystems Dragon). Results were indicated in percentage of hemolytic inhibition. Inhibition of lysis (%) = 100 − 100 × (OD_sample_ − OD_sample  background_) ÷ OD_100%  lysis_.

### 2.4. Ultra High Performance Liquid Chromatography-Mass Spectrometry (UHPLC-MS) Analysis of RJFs

The UHPLC was performed on an Agilent 6540 accurate-mass Q-TOF LC/MS system (Agilent Technologies, USA). A UPLC C18 analytical column (2.1 mm × 100 mm, I.D. 1.7 *μ*m, ACQUITY UPLC BEH, Waters, USA) was conducted for separation, coupled with a C18 precolumn (2.1 mm × 5 mm, I.D. 1.7 *μ*m, Van Guard TM BEH, Waters, USA) at room temperature of 20°C. The mobile phase was a mixture of water (A) and acetonitrile (B), both containing 0.1% formic acid, using a gradient elution (0 min: 5% B, 8 min: 25% B, 18 min: 75% B, 25 min: 100% B, 28 min: 100% B) with 3 min of balance back to 5% B. The injection volume was 1 *μ*L, and the flow rate was set at 0.4 mL/min. Mass spectra were obtained in positive mode, and the source parameters were set as follows: Gas Temp 300°C, Gas Flow 8 L/min, Nebulizer 40 psi, Vcap 3500, Nozzle Voltage (*V*) 500, and Fragmentor 120. Reference masses were used at* m/z *121.0509 (purine) and 922.0098 (hexakis phosphazine).

Reference compounds (>95% by HPLC) isolated in our laboratory were solved in pure methanol solution, and the concentration of each standard solution was made as follows: quercetin 336 *μ*g/mL, luteolin 308 *μ*g/mL, vitexin 143 *μ*g/mL, isoquercitrin 336 *μ*g/mL, and GLA 500 *μ*g/mL. Then a stock solution of mixed reference compounds was prepared for calibration, containing 67.2 *μ*g/mL quercetin, 61.6 *μ*g/mL luteolin, 28.6 *μ*g/mL vitexin, 67.2 *μ*g/mL isoquercitrin, and 100.0 *μ*g/mL GLA.

Data analysis was performed on Agilent MassHunter Workstation software-Qualitative Analysis (version B.04.00, Build 4.0.479.5, Service Pack 3, Agilent Technologies, Inc. 2011). It was applied in positive ion mode with the following settings: extraction restrict retention time of 2–20 min, peaks with height ≥100 counts to be used, charge state of 1, and peak spacing tolerance of 0.0025* m/z*, plus 5.0 ppm; compound relative height ≥2.5%, and absolute height ≥1000 counts; elements of C, H, O, N, from 3–60, 0–120, 0–30, 0–30, respectively, to generate formulae. Results are shown by base peak chromatogram (BPC with* m/z* range 100–950).

### 2.5. The Contents of Glaucocalyxin A (GLA), Quercetin, and Luteolin in RJFs by High Performance Liquid Chromatographic (HPLC) Analysis

HPLC quantitative analysis was performed using an Agilent Infinity 1260 system with a YMC C18 column (4.6 × 250 mm, 5 *μ*m). The HPLC was performed with the mobile phase, which was a mixture of 50% water (A) and 50% methanol (B), both containing 0.1% formic acid. The injection volume was 20 *μ*L and the flow rate was set at 1.0 mL/min, and peaks were monitored at 230 and 365 nm. Calibration curves consisted of 0.064–2.001 mg/mL GLA, 0.063–2.206 mg/mL quercetin, and 0.063–2.006 mg/mL luteolin standard solutions. Peaks were identified by congruent retention times compared with standards. Analyses were performed in triplicate.

### 2.6. Determination of Total Flavonoid Content in RJFs

Total flavonoids in RJFs were determined using a slightly modified colorimetric method described previously [15]. A 13 mL methanol of diluted sample solution was mixed with 1 mL of a 5% NaNO_2_ solution. After 6 min, 1 mL of a 10% AlCl_3_ solution was added and allowed to stand for 6 min then 10 mL of a 4% NaOH solution was added to the mixture and left to stand for another 15 min. The absorbance of the mixture was determined at 510 nm, and luteolin was used as the standard.

### 2.7. Animals

Male Kunming mice, about 24–28 g, were purchased from the Center of Experimental Animals Soochow University (Suzhou, Jiangsu, China). The mice were kept in a specific pathogen free condition and received food and water* ad libitum*. Laboratory temperature was 24 ± 1°C, and relative humidity was 40–80%. Before experimentation, the mice were allowed to adapt to the experimental environment for a minimum of 3 days. The experimental protocols shown in this study were approved by the Animal Ethical Committee of School of Pharmacy at Soochow University.

### 2.8. Establishment of the ALI Model and Preventive Regimen [16–24]

RJFs were ground and suspended in distilled water containing 0.5% sodium carboxymethyl cellulose (CMC-Na) for administration to mice.

The mice were randomly divided into seven groups (*n* = 20): control group, RJFs group (RJFs treated at 25.6 mg/kg without LPS), model (LPS, 2 mg/kg) group, RJFs-pretreated groups (RJFs at 6.4, 12.8, and 25.6 mg/kg with LPS, resp.), and positive group (DXM, 5 mg/kg).

RJFs and RJFs-pretreated groups received an intragastric injection of RJFs at given doses, each mouse was administered orally once per day for 7 days, consecutively. Positive group received DXM only on day 7. Mice from control, model, and positive groups received the equal volume distilled water instead of RJFs. The doses of these drugs we chose were on the basis of our preliminary experiments. On day 7, 2 h after RJFs and DXM treatment, mice were slightly anesthetized with a 20% urethane (4 mL/kg) given intraperitoneally. Then, in experimental groups, 2 mg/kg LPS was instilled intratracheally (i.t.) in 50 *μ*L NS to induce lung injury. Mice from control and RJFs groups were given a 50 *μ*L NS i.t. instillation without LPS. It took about 5-6 min per mouse to induce lung injury. Animals recovered quickly from the procedure with only mild discomfort.

6 h after LPS challenge, half of the mice of each group (*n* = 10) were sacrificed and the blood samples were collected (each one was approximately 1 mL). The right lung was used to collect BALF, which was lavaged three times with 0.8 mL of autoclaved NS. The left lung was used to obtain the lung W/D ratio. 24 h after LPS challenge, the rest of the mice were sacrificed. The inferior lobe of right lung was used for histopathologic evaluation. The superior lobes of right lung were used to collect BALF. The left lung was homogenized using a homogenizer.

### 2.9. NO and Protein Analysis

BALF was collected as previously described. At 6 h after LPS challenge, mice were sacrificed by exsanguination. BALF was obtained by intratracheal instillation, each sample was centrifuged (4°C, 1400 ×g, 10 min) and its supernatants were stored at −80°C for analysis of NO and protein levels. The content of NO and protein in the supernatants of the BALF (6 h) were measured by NO and BCA protein assay kits according to the manufacturer's instructions strictly.

### 2.10. Lung W/D Ratio

Mice were sacrificed by exsanguination at 6 h after LPS challenge. The left lung was excised, blotted dry, and weighed to obtain the “wet” weight and then placed in an oven at 80°C for 48 h to obtain the “dry” weight. The ratio of the wet lung to the dry lung was calculated to assess tissue edema.

### 2.11. Complement 3 (C3) in Serum Analysis

At 6 h after LPS challenge, the blood of mice was collected. Blood samples were coagulated at room temperature for 10 min and then centrifuged (4°C, 1400 ×g, 20 min), its supernatants (serum) were stored at −80°C for subsequent analysis. The level of complement 3 in serum was quantified by the sandwich ELISA kit, according to the manufacturer's instructions strictly.

### 2.12. Assays for SOD and MPO Activities

At 24 h after LPS challenge, the left lungs were homogenized using a homogenizer, then they were prepared to 10% lung tissue homogenate. The tissue homogenate generated was assayed for MPO activity, which was measured by MPO determination kit using commercially available reagents according to the manufacturer's instructions. The homogenate was then centrifuged at 1400 ×g for 10 min at 4°C. The supernatants obtained were used for assay of SOD activity. SOD activity was expressed as units per milligram of protein.

### 2.13. Cytokine Analysis

The levels of cytokine TNF-*α*, IL-6, and IL-1*β* in the supernatants of the BALF (24 h) were quantified in duplication by the sandwich ELISA kit using commercially available reagents, according to the manufacturer's instructions strictly.

### 2.14. Histological Studies of Lung

After mice were sacrificed, inferior lobe of right lung tissue was immediately harvested and fixed in 4% formaldehyde. Then lung tissue was dehydrated and embedded in paraffin. Paraffin sections were stained with hematoxylin and eosin (H&E) according to the regular staining method. Images were observed under an Olympus microscope at an original magnification of 400x.

For the detection of complement deposits, the 5 *μ*m sections were deparaffinized, rehydrated, and incubated with rabbit anti-human C3c overnight at 4°C. Slices were visualized using chromogenic substrate solution 3,3′-diaminobenzidine (DAB). All slides were imaged with CoolPix 4500 camera (Nikon) matched on a CX21 microscope (Olympus) at an original magnification of 400x.

### 2.15. Statistical Analysis

All values were expressed as mean ± S.D. To perform statistical analysis, one-way analysis of variance (ANOVA) was used. If any significant changes were found, post hoc comparisons were performed using Fisher's PLSD. Statistical significance was accepted at *P* < 0.05.

## 3. Results

### 3.1. Chemical Profiling of RJFs

**Figure 1:**
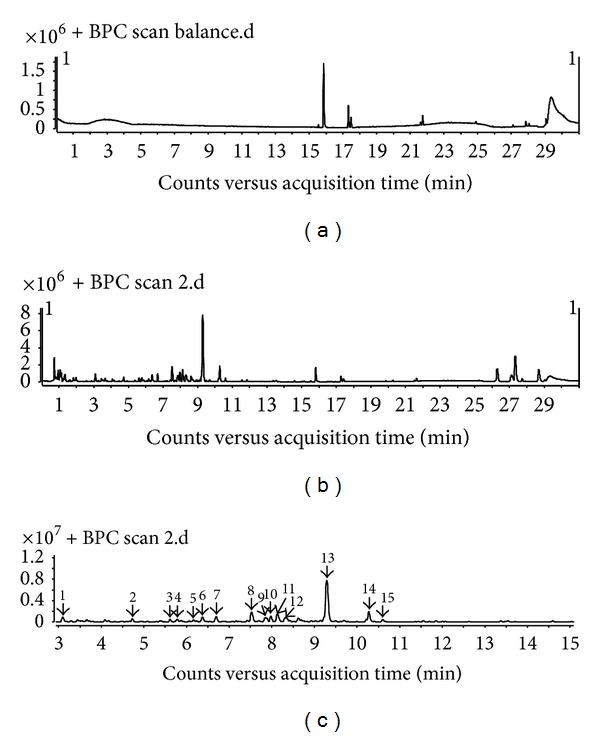
LC-MS base peak chromatograms of RJFs. The peak numbers referred to Table 1. (a) Methanol. (b) RJFs (0–30 min). (c) RJFs (3–15 min).

A previous study has demonstrated that* Rabdosia japonica* var.* glaucocalyx* (Maxim.) Hara primarily contains flavonoids and terpenoids [11–13]. AB-8 macroporous adsorption resins were used for flavonoid and diterpenoids enrichment of RJFs. By UHPLC-Q-TOF-MS analysis, a total of 15 well-separated chromatographic peaks in RJFs could be found in the BPC of RJFs (Figure 1). In LC-MS analysis, five peaks can be achieved via comparison of the reference standards' retention data and MS spectra. Other detected peaks were tentatively identified by their accurate mass data in comparison with reported references. RJFs mainly contain flavonoid compounds. As shown in Table 1, peaks 3, 4, 5, 6, 7, 8, 12, 13, and 15 were tentatively identified to be flavonoids and peaks 9 and 14 were tentatively identified to be terpenoids. In our previous study, we had isolated some compounds such as peaks 1, 3, 5, 9, 13, 14, and 15 [11–13]. The results indicated that the GLA, quercetin, and luteolin contents were 2.90%, 0.308%, and 0.291% of RJFS, respectively, by HPLC analysis. The total flavonoids content were 12.90% of RJFs by colorimetric method.

### 3.2. In Vitro Anticomplementary Activity of RJFs

**Figure 2:**
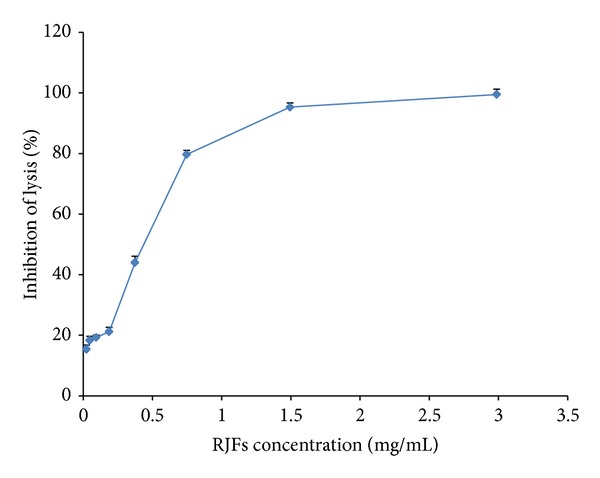
Inhibition of the classical pathway-mediated hemolysis by RJFs. Lysis of sheep erythrocytes in 1 : 60 diluted Guinea pig serum in the presence of increasing amounts of RJFs. Data are listed as mean ± S.D. (*n* = 3).

As shown in Figure 2, RJFs significantly inhibited hemolysis in sheep erythrocytes induced by Guinea pig serum (CP_50_ = 0.354 ± 0.066 mg/mL).

### 3.3. Effects of RJFs on Lung W/D Ratio, NO, and Total Protein Concentration in BALF of Mice 

**Figure 3:**
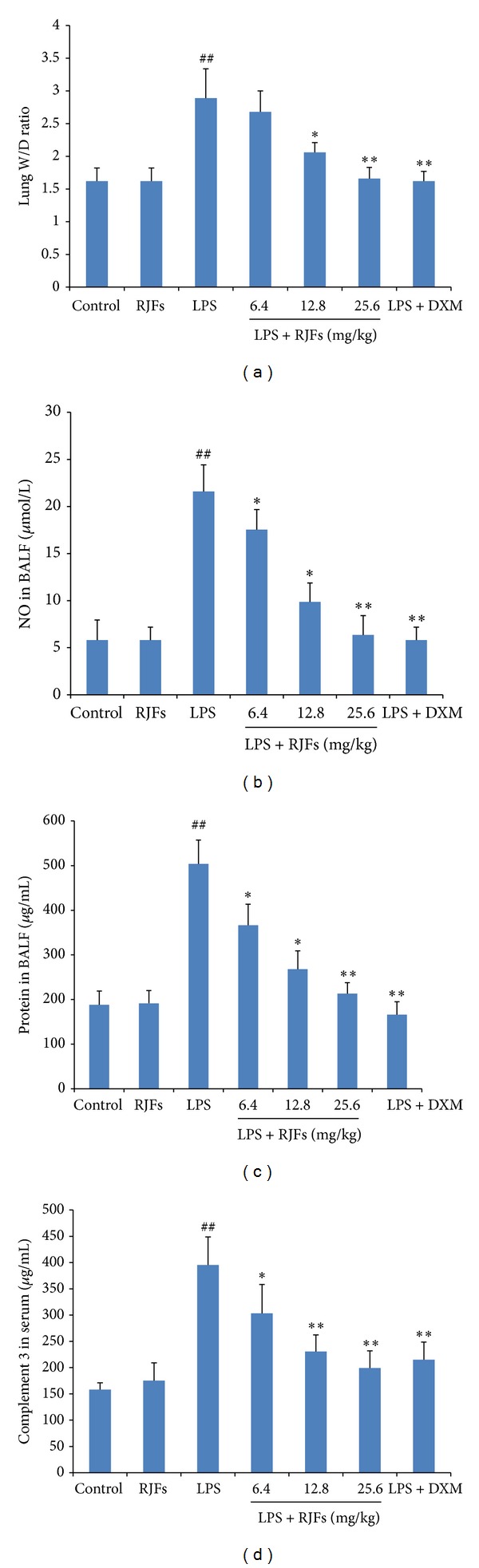
Effects of RJFs on the lung W/D ratio, NO, total protein concentration in BALF, and the level of C3 in serum of mice. 2 h after RJFs pretreatment on day 7, mice were slightly anesthetized, and then 2 mg/kg LPS was instilled intratracheally (i.t.) to induce lung injury. The lung W/D ratio was determined at 6 h after LPS challenge; BALF and the serum were collected at 6 h following LPS challenge. NO and total protein concentration in the supernatant of BALF were determined by kits. C3 activity in serum was determined by mouse C3 ELISA kit. Data expressed as means ± S.D. (*n* = 10); ^##^ *P* < 0.01 compared with control, *P < 0.05 and **P < 0.01 compared with vehicle treated ALI model group.

As shown in Figure 3, the lung W/D ratio, NO, and the total protein concentration in BALF were found to be significantly higher after LPS challenge compared with those of the control group (*P* < 0.01), while RJFs (25.6 mg/kg) gavage itself did not cause significant changes.

Compared with vehicle treated ALI model group, pretreatment with RJFs markedly decreased the NO and the total protein concentration (*P* < 0.05) in BALF of mice (Figures 3(b) and 3(c)). RJFs at 12.8 and 25.6 mg/kg significantly decreased the lung W/D ratio (*P* < 0.05) (Figure 3(a)). DXM also significantly decreased the lung W/D ratio, NO, and the total protein concentration.

### 3.4. Effect of RJFs on C3 Production in Serum of LPS-Induced ALI

As shown in Figure 3(d), the level of C3 was significantly higher after LPS challenge compared with those of the control group (*P* < 0.01), while RJFs (25.6 mg/kg) gavage itself did not cause significant changes. Compared with vehicle treated ALI model group, pretreatment with RJFs markedly decreased the level of C3 (*P* < 0.05) in serum of mice. RJFs at 12.8 and 25.6 mg/kg significantly decreased the level of C3 (*P* < 0.01). DXM also significantly decreased the level of C3.

### 3.5. Effects of RJFs on SOD and MPO Activities in Lung Tissues of LPS-Induced ALI

**Figure 4:**
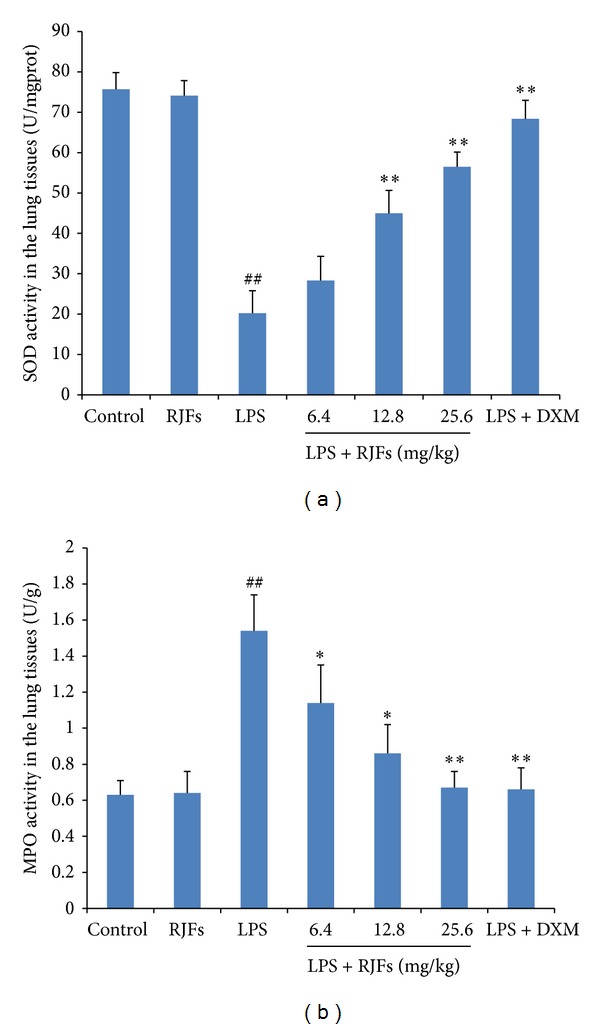
Effects of RJFs on SOD and MPO activities of lung tissue in mice. 2 h after RJFs pretreatment on day 7, mice were slightly anesthetized, and then 2 mg/kg LPS was instilled intratracheally (i.t.) to induce lung injury. Lung homogenates were prepared at 24 h after LPS challenge. SOD and MPO activities were determined by kits. Data expressed as means ± S.D. (*n* = 10); ^##^ *P* < 0.01 compared with control, *P < 0.05 and **P < 0.01 compared with vehicle treated ALI model group.

As shown in Figure 4, LPS challenge resulted in significant decreases of SOD activity and significant increases of MPO activity in the lungs compared with the control group (*P* < 0.01), while RJFs (25.6 mg/kg) gavage itself did not cause significant changes.

RJFs at 12.8 and 25.6 mg/kg significantly increased SOD activity (*P* < 0.01) (Figure 4(a)), while RJFs at 25.6 mg/kg decreased MPO activity (*P* < 0.01) (Figure 4(b)). DXM also significantly increased SOD activity and decreased MPO activity.

### 3.6. Effects of RJFs on TNF-*α*, IL-6, and IL-1*β* Production in BALF

**Figure 5:**
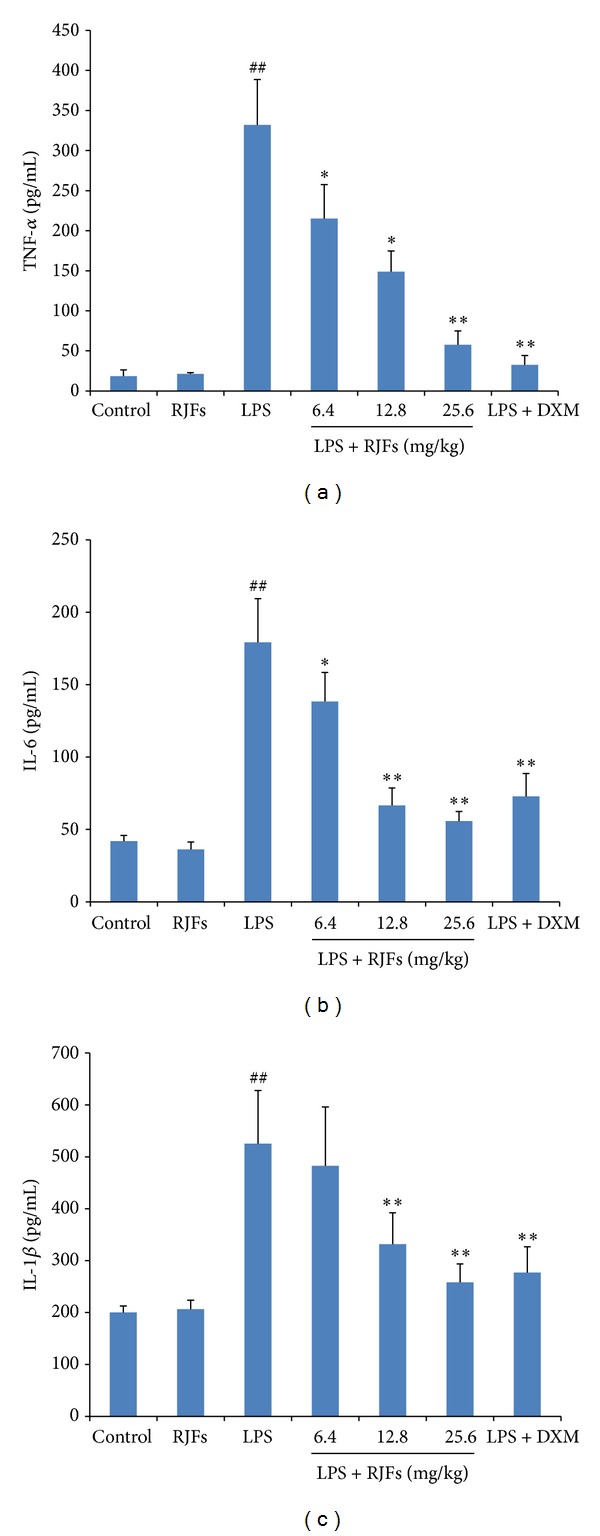
Effects of RJFs on production of inflammatory cytokine TNF-*α*, IL-6, and IL-1*β* in BALF of mice. 2 h after RJFs pretreatment on day 7, mice were slightly anesthetized, and then 2 mg/kg LPS was instilled intratracheally (i.t.) to induce lung injury. BALF was collected at 24 h following LPS challenge. The levels of TNF-*α*, IL-6, and IL-1*β* in the supernatants of BALF were determined by mouse ELISA kits. Data expressed as means ± S.D. (*n* = 10); ^##^ *P* < 0.01 compared with control, *P < 0.05 and **P < 0.01 compared with vehicle treated ALI model group.

As shown in Figure 5, LPS significantly increased TNF-*α*, IL-6, and IL-1*β* production compared with the control group (*P* < 0.01), while RJFs (25.6 mg/kg) gavage itself did not cause significant changes.

Compared with vehicle treated ALI model group, pretreatment with RJFs markedly decreased the level of TNF-*α* in the BALF of mice (*P* < 0.05) (Figure 5(a)). RJFs at 12.8 and 25.6 mg/kg significantly reduced the level of IL-6 and IL-1*β* in the BALF (*P* < 0.01) (Figures 5(b) and 5(c)). DXM also significantly decreased TNF-*α*, IL-6, and IL-1*β* production.

### 3.7. Effect of RJFs on Lung Histology

**Figure 6:**
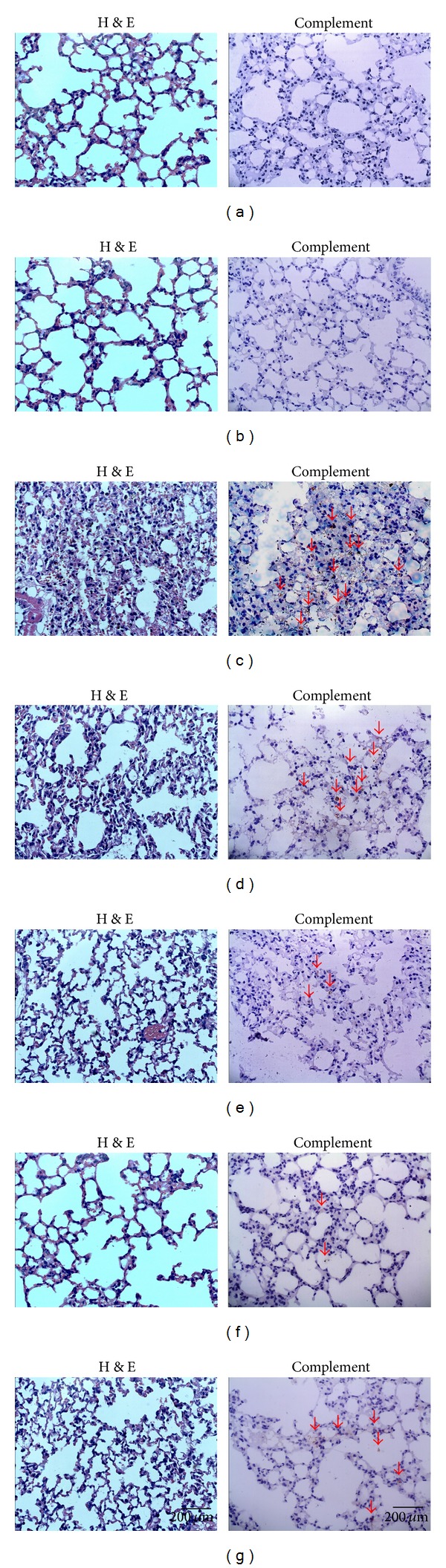
Hematoxylin and eosin-stained (H&E) (400x) and immunohistochemistry of lung tissue (400x). Lungs from each group were processed for histological evaluation at 24 h after LPS challenge: Section of control (a) and RJFs (b) groups mice: normal lung tissue sections (H&E and complement). Section of a LPS-induced ALI model (c) group mouse: note increased alveolar wall thickness, inflammatory cells aggregation, pulmonary hemorrhage (H&E), and a patchy dense immunoperoxidase indicative of complement deposition marked with red arrows (complement). Section from 6.4, 12.8, and 25.6 mg/kg RJFs-pretreated ((d), (e), and (f), resp.) and DXM-treated (g) groups mouse: note mild alveolar wall thickness, reduced inflammatory cells aggregation, little pulmonary hemorrhage (H&E), and little complement deposition marked with red arrows (complement).

Histopathological changes of each group were observed by histochemical staining with H&E. Histopathological changes such as lung edema, increased alveolar wall thickness, inflammatory cells aggregation, and pulmonary hemorrhage were observed in model group mice induced by LPS given intratracheally. As shown in Figure 6, these lesions were not apparent in control group stimulated with only NS. Pretreatment with RJFs markedly ameliorated the pulmonary injury.

For vehicle treated ALI model group, immunohistochemistry of lung tissue sections showed a patchy dense immunoperoxidase indicative of complement deposition. Complement appeared by bulk deposition and was mainly deposited in lung tissue. In contrast, mice in control group had little complement deposition in lung tissue. Pretreatment with RJFs markedly decreased complement deposition (Figure 6).

## 4. Discussion

*Rabdosia japonica *var.* glaucocalyx* (Maxim.) Hara was used as a folk medicine for a long time and contained mainly diterpenoids, flavonoids, and steroids [11–13]. In our previous study, we found the flavonoids (such as quercetin, luteolin, isoquercetin, rutin, etc.) and the phenylpropionic acid (such as caffeic acid and caffeic acid ethylene ester) have anticomplementary activity in the test of complementary activity through the classical pathway, but the diterpenoids (such as GLA) did not have anticomplementary activity [13]. Therefore, RJFs were prepared for study and the results showed it exhibited a high anticomplementary activity.

The UHPLC-Q-TOF-MS analysis indicated RJFs had mainly flavonoids, fewer terpenoids, and the phenylpropionic acid. In this study, we demonstrated that RJFs significantly alleviated the LPS-induced ALI in mice by reducing the production of inflammatory mediators and downregulation of the complement level.

LPS, a large molecule consisting of a lipid and a polysaccharide joined by a covalent bond, is the major component of the outer membranes of Gram-negative bacteria, acts as endotoxins, and elicits strong inflammatory and immune response in animals [18, 19]. It is an important inducer of lung injury which can be employed in the investigation of ALI [20–22]. Therefore, an animal model of direct ALI was established and used by i.t. instillation of LPS in mice in our study. Glucocorticoids can cause neutrophilic granulocyte and alveolar macrophages, that are used to alleviate respiratory in the clinical treatment of ALI/ARDS [23, 24]. Therefore, DXM was used as a positive control to evaluate the anti-inflammatory efficiency of RJFs in LPS-induced ALI.

In our present investigation, we evaluated the lung wet-to-dry weight ratio to quantify the magnitude of pulmonary edema, which is a typical symptom of inflammation and a major characteristic of ALI [1, 25, 26]. Here, RJFs decreased the lung W/D ratio, which indicated that RJFs could inhibit the leakage of serous fluid into lung tissue and attenuate the development of pulmonary edema. As other indexes of epithelial and endothelial permeability in ALI, we measured the total protein content in the BALF [27]. As expected, mice exposed to LPS presented high protein content in the BALF, which were inhibited by RJFs.

NO has well been demonstrated in the pathophysiology and development of ALI induced by LPS, although its role in the pathogenesis of ALI has not been clearly elucidated [28]. LPS is well demonstrated to stimulate iNOS expression and NO overproduction in many pulmonary cell types, including lung endothelial cells, epithelial cells, macrophages, and neutrophils [29]. In our experiment, RJFs reduced the productions of NO in BALF, which might contribute to the amelioration of inflammatory response and the lessened lung damage.

Oxidative stress plays an important role in the development of LPS-induced ALI. MPO was released by neutrophils, which could act as an index to reflect the activation, adhesion, and recruitment of neutrophils into lung [30]. Neutrophils excrete MPO in the extracellular medium, bringing about an accumulation of H_2_O_2_-Cl and several other reactive oxygen derivatives and leading to an oxidative modification of proteins or cellular structures [31]. The present study showed that LPS induced a significant enhancement of MPO activity in mice lung parenchyma after LPS challenge in comparison to control mice, indicating a significant recruitment of neutrophils in lung parenchyma. RJFs inhibited pulmonary parenchymal MPO activity and were consistent with decreased number of neutrophils in the BALF, suggesting a mechanism by which RJFs inhibited LPS-induced ALI. SOD is an enzyme that exists in cells removing oxyradicals, whose activity variation may represent the degree of tissue injury [32]. In this study, RJFs enhanced SOD activity, suggesting RJFs may effectively scavenge oxyradicals during the inflammatory response to LPS-induced ALI.

LPS is known to induce the production of several inflammatory and chemotactic cytokines. TNF-*α*, which mainly originate from macrophages, damage vascular endothelial cells and increase their permeability, resulting in leukocyte adhesion, granulocyte degranulation, leukocyte migration into inflammatory positions, and lung lesion [33, 34]. Proinflammatory cytokines TNF-*α*, IL-6, and IL-1*β* appear in the early phase of an inflammatory response, play a critical role in the pathophysiology of inflammation in ALI, and contribute to the severity of lung injury [22, 35]. High levels of TNF-*α*, IL-6, and IL-1*β* in the BALF have been noted in patients with ALI/ARDS, and the persistent elevation of proinflammatory cytokines in humans with ALI or sepsis has been associated with more severe outcomes [36]. According the changes of inflammatory mediators in BALF and serum following intrapulmonary challenge with LPS in our previous studies, we chose to detect the inflammatory mediators in BALF at 24 h. In the present study, LPS induced the production of large amounts of TNF-*α*, IL-6, and IL-1*β* in the BALF of mice; RJFs lowered TNF-*α*, IL-6, and IL-1*β* secretion at 24 h after LPS challenge. Therefore, RJFs may protect against LPS-induced ALI by decreasing the production of these proinflammatory cytokines.

The levels of TNF-*α*, IL-6, and IL-1*β* are regulated by the activation of transcription factor NF-*κ*B, which plays a crucial role in immune and inflammatory responses [37, 38]. It reported that total flavonoids of* Mosla scabra* (MF) leaves could attenuate pulmonary inflammation in mice with LPS-induced ALI, and the protective effect of MF in ALI might be related to its suppression of NF-*κ*B and MAPK activation and, subsequently, caused a remarkable reduction in inflammatory cell infiltration and inflammatory cytokine secretion in lung tissues [39]. Also, luteolin suppresses inflammatory mediator expression by blocking the Akt/NF*κ*B pathway in acute lung injury induced by lipopolysaccharide [40]. Moreover, luteolin attenuates the pulmonary inflammatory response which involves abilities of antioxidation and inhibition of MAPK and NF-*κ*B pathways in mice with endotoxin-induced acute lung injury in mice [41]. The most important fraction of the RJFs is flavonoid, so the potential mechanisms of flavonoid in the treatment of acute lung injury are also associated with NF-*κ*B pathways possibly.

Activation of the complement system plays a key role in normal inflammatory response to injury but may cause substantial injury when activated inappropriately. The complement system consists of more than 30 serum and cellular proteins, including positive and negative regulators, linked in three biochemical cascades, the classical, alternative and lectin complement pathways [42, 43]. C3 activation leads to the entry of the final common pathway resulting in the formation of the membrane attack complex (MAC, C5b-9) [44]. Animal model of ALI studies demonstrated alterations of complement 3 levels in ALI [5]. Pretreatment of wild type mice with humanized cobra venom factor, which inactivates C3, decreased polymorphonuclear neutrophil (PMN) in BAL cells and reduces C3 deposition in the lung [4]. Therefore, complement 3 plays an important role in activating complement system of ALI. As we detected the changes of C3-level in serum following intrapulmonary challenge with LPS, we found that it increased significantly at 6 h. In this research, the level of C3 in serum increased obviously at 6 h after LPS challenge; then, at 24 h, immunohistochemistry of lung tissue sections showed overactivation of complement with abundantly complement deposition in vehicle treated ALI model group. RJFs markedly decreased complement deposition and the level of C3 in serum which might contribute to the attenuation of lung injury. We thought that inhibitors of complement may be potential adjunctive treatments for LPS-induced ALI.

*Rabdosia japonica *var.* glaucocalyx* (Maxim.) Hara is used as traditional medicinal herb for centuries in China with low toxicity [7]. In this study, RJFs (25.6 mg/kg) gavage itself did not cause significant changes; therefore, RJFs are effective with low toxicity, indicating them as a potential therapeutic agent for ALI.

## 5. Conclusion

In conclusion, RJFs attenuated LPS-induced lung injury, including reduction of lung W/D ratio, inhibition of protein level, and NO overproduction in BALF. In addition, RJFs lowered TNF-*α*, IL-6, and IL-1*β* level in BALF in ALI mice. The increased SOD activity and inhibited MPO activity in lung tissue were also observed in RJFs-pretreated ALI mice. Histological examination showed that RJFs significantly ameliorated lung injury by improving lung morphology and decreasing complement deposition. Meantime, RJFs obviously reduced the level of C3 in serum in ALI mice. The effects of RJFs against ALI were related with the inhibition on the production of proinflammatory mediators and decreasing the level of complement.

## Figures and Tables

**Table 1:** Characteristics of chemical components of RJFs by UHPLC-Q-TOF-MS.

Peaks number	Retention time (min)	Mass ions (*m*/*z*)	Molecular formula	Identification
1	3.104	198.0755 [M + H_2_O]^+^	C_9_H_8_O_4_	Caffeic acid
2	4.731	351.1248 [M + H]^+^	C_20_H_30_O_5_	RabdoloxinB
3	5.882	434.2496 [M + 2H]^+^	C_21_H_20_O_10_	Vitexin
4	5.915	451.2321 [M + H + H_2_O]^+^	C_21_H_20_O_10_	Isovitexin
5	6.240	437.1120 [M + H–CO]^+^	C_21_H_20_O_12_	Isoquercitrin
6	6.374	317.2116 [M–H]^+^	C_15_H_10_O_8_	Myricetin
7	6.691	283.7185 [M–H]^+^	C_16_H_12_O_5_	Baicalein-methylether
8	7.525	340.2600 [M–H + Na]^+^	C_15_H_10_O_8_	Quercetagetin
9	7.859	351.2127 [M + H]^+^	C_20_H_30_O_5_	Kamebakaurin
10	7.975	745.3335 [2M + Na]^+^	C_18_H_16_O_7_	Methyl-6-dehydroxyl rosmarinate
11	8.125	396.8057 [M + Na–H]^+^	C_19_H_18_O_8_	Methyl rosmarinate
12	8.317	426.2526 [M + H + Na]^+^	C_20_H_18_O_9_	Apigenin-7-arabinoside
13	9.293	285.1869 [M–H]^+^	C_15_H_10_O_6_	Luteolin
14	10.277	333.2062 [M + H]^+^	C_20_H_28_O_4_	Glaucocalyxin A
15	10.611	785.4507 [2M–H + 2HCOONa + 2Na]^+^	C_15_H_10_O_7_	Quercetin

## Abbreviations

RJ: * Rabdosia japonica *var*. glaucocalyx* (Maxim.) Hara
RJFs: The Flavonoids Fraction of* Rabdosia japonica* var.* glaucocalyx* (Maxim.) Hara
LPS: Lipopolysaccharide
ALI: Acute Lung Injury
BALF: The Bronchoalveolar Lavage Fluid
C3: Complement 3
ELISA: Enzyme Linked Immunosorbent Assay
Lung W/D ratio: Lung Wet-to-Dry Weight Ratio
MPO: Myeloperoxidase
SOD: Superoxide Dismutase
BCA: Bicinchoninic Acid
ARDS: Acute Respiratory Distress Syndrome
DXM: Dexamethasone
NO: Nitric Oxide
VBS: Veronal Buffer Saline
EAs: Sensitized Erythrocytes
H&E: Hematoxylin and Eosin
GLA: Glaucocalyxin A
BPC: Base Peak Chromatograms
UHPLC-MS: Ultra High Performance Liquid Chromatography-Mass Spectrometry
Q-TOF-MS: Quadrupole Time of Flight Tandem Mass Spectrometry.
